# Clean Air, Smart Cities, Healthy Hearts: Action on Air Pollution for Cardiovascular Health

**DOI:** 10.5334/gh.1073

**Published:** 2021-09-07

**Authors:** Michael Brauer, Narantuya Davaakhuu, Maria Consuelo Escamilla Nuñez, Michael Hadley, Daniel Kass, Mark Miller, Dorairaj Prabhakaran, Karen Sliwa, Ta-Chen Su, Ilonca C. H. Vaartjes, Rajesh Vedanthan, Jeremiah Mwangi, Kelcey Armstrong-Walenczak

**Affiliations:** 1 The University of British Columbia, Institute for Health Metrics and Evaluation, University of Washington, CA; 2 University of Washington, US; 3 National Center for Public Health Mongolia, MN; 4 Instituto Nacional de Salud Pública, MX; 5 Mount Sinai, US; 6 Vital Strategies, US; 7 Centre for Cardiovascular Sciences, University of Edinburgh, UK; 8 Public Health Foundation India, IN; 9 Cape Heart Institute, Faculty of Health Sciences, University of Cape Town, ZA; 10 Department of Environmental and Occupational Medicine, National Taiwan University College of Medicine, TW; 11 Julius Center for Health Sciences and Primary Care, University Medical Center Utrecht, NL; 12 NYU Grossman School of Medicine, New York, US; 13 World Heart Federation, CH

**Keywords:** air pollution, cardiovascular disease, cvd, environmental health, climate change, policy

## Abstract

More than twenty percent of all cardiovascular disease (CVD) deaths are caused by air pollution — more than three million deaths every year — and these numbers will continue to rise unless the global community takes action. Nine out of ten people worldwide breathe polluted air, which disproportionately affects those living in low-resource settings. The World Heart Federation (WHF) is committed to reducing the impact of air pollution on people’s health and has made this a priority area of its global advocacy efforts. In pursuit of this goal, WHF has formed an Air Pollution Expert Group to inform action on air pollution for CVD health and recommend changes to public health policy. This policy paper lays out the health impacts of air pollution, examines its position on the global policy agenda, demonstrates its relevance to the cardiovascular community, and proposes actionable policy measures to mitigate this deadly risk factor to health. The paper considers the important roles to be played by the Members of WHF, including scientific societies and the physicians that constitute them, heart health foundations, and patient advocacy groups. The paper concludes with a detailed table of recommendations for the various sub-target groups at the global, national, local, and patient level.

**Figure:**
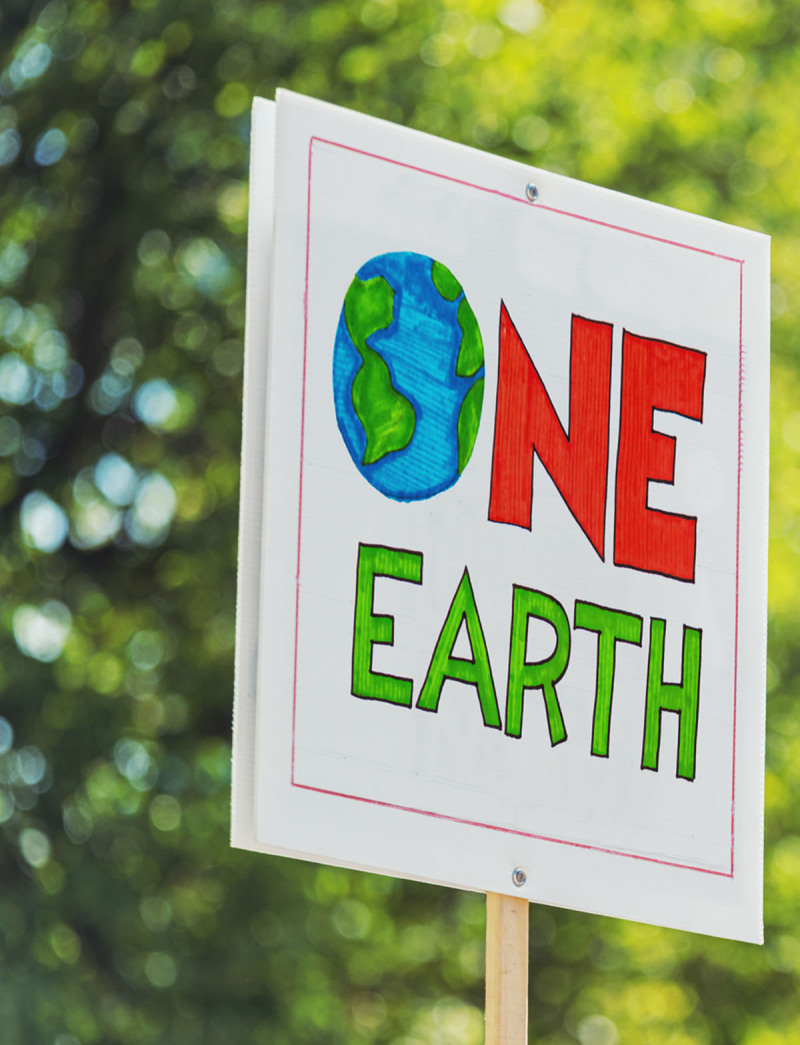


## Executive Summary

**More than twenty percent of all cardiovascular disease (CVD) deaths are caused by air pollution — more than three million deaths every year — and these numbers will continue to rise unless the global community takes action. Nine out of ten people worldwide breathe polluted air, which disproportionately affects those living in low-resource settings.**

The World Heart Federation (WHF) is committed to reducing the impact of air pollution on people’s health and has made this a priority area of its global advocacy efforts. In pursuit of this goal, WHF has formed an Air Pollution Expert Group to inform action on air pollution for CVD health and recommend changes to public health policy.

This policy paper lays out the health impacts of air pollution, examines its position on the global policy agenda, demonstrates its relevance to the cardiovascular community, and proposes actionable policy measures to mitigate this deadly risk factor to health. The paper considers the important roles to be played by the Members of WHF, including scientific societies and the physicians that constitute them, heart health foundations, and patient advocacy groups. The paper concludes with a detailed table of recommendations for the various sub-target groups at the global, national, local, and patient level.

**Figure:**
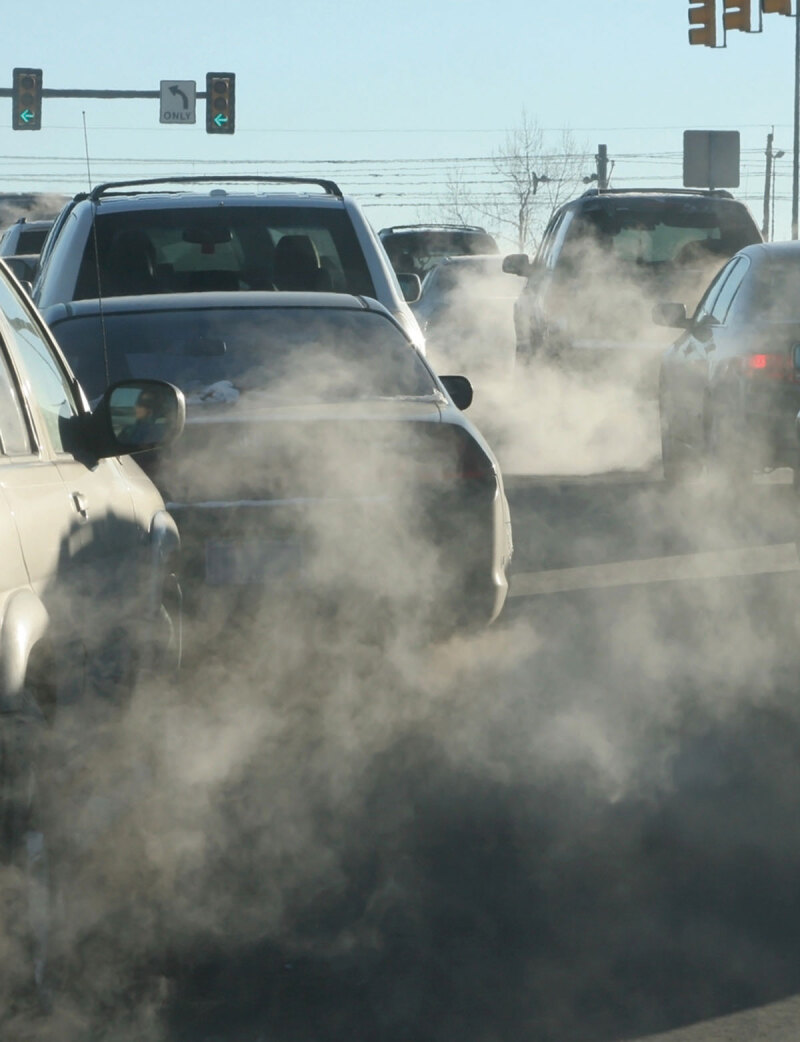


## Air pollution and human health – a tale as old as time

**In October 2018, World Health Organization (WHO) Director-General Dr Tedros Adhanom Ghebreyesus made headlines around the world by declaring that “No one, rich or poor, can escape air pollution. It is a silent public health emergency”** [1].

His statement, together with the prominence of the first-ever WHO Global Conference on Air Pollution and Health that it was published to promote, rendered the issue ‘silent’ no more. Seemingly overnight, air pollution was propelled to the top of the global public health agenda. For three days, a diverse and high-level group of Member State representatives, major city mayors, non-governmental organizations, and academics presented the latest findings linking air pollution and ill health effects. The conference concluded with a strong commitment from participants to advocate for a reduction in air polluting climate emissions and strive towards achieving WHO air quality guidelines [2].

While there has been a marked surge of attention in recent years, the health impacts of air pollution have been observed, measured, and to some extent mitigated for centuries. From citizen complaints about cooking smoke in the capital of the Holy Roman Empire to committees fighting to regulate the smog-producing factories of the Industrial Revolution, humans have been working to avoid or reduce the detrimental effects of polluted air for nearly as long as they have been gathering in permanent settlements [3]. Nevertheless, formal policies to limit pollutant-producing industry and various ‘Clean Air’ acts did not gain significant traction in many countries until the mid-1900s, and it is only in recent times that policymakers have started granting prominence to the specific health impacts of man-made pollution at the global level. Deeper scientific understanding of the role of air pollution in human health continues to develop, and appreciation for air pollution as a CVD risk factor is relatively new. Growing environmental protection movements around the world, increasing legal recognition for the health impacts of air pollution (such as the landmark inquest into the death of Ella Adoo-Kissi-Debrah which ruled that air pollution contributed to her death) the adoption of the cross-cutting Sustainable Development Goals, the agenda-setting High-Level Meetings on Non-Communicable Diseases (NCDs), and the subsequent Conference on Air Pollution and Health have together created an unprecedented momentum to act on this critical public health issue [4].

**Figure:**
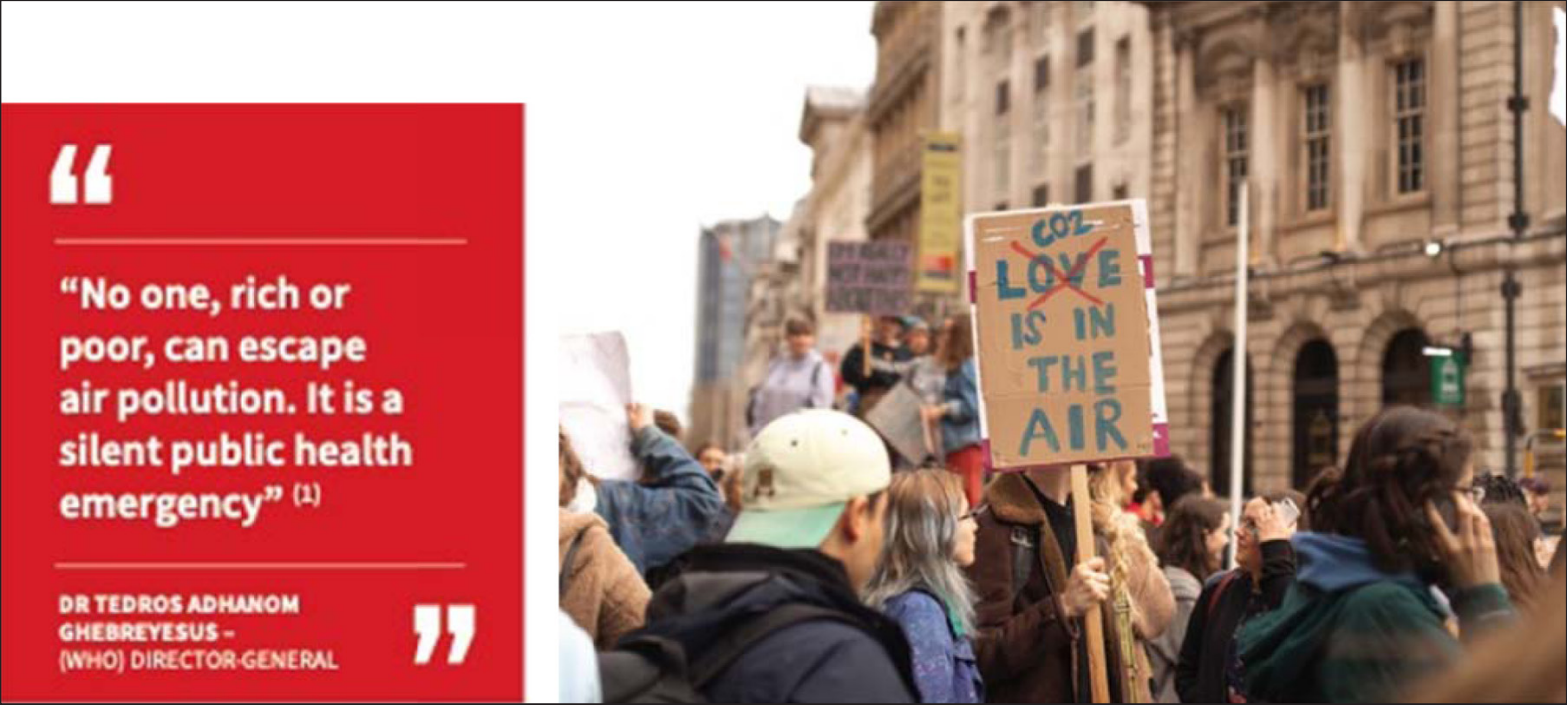
Shaw C. Unsplash; 2019 [5].

## What is air pollution?

Air pollution is a complex and dynamic mixture of numerous compounds in gaseous and particle form, originating from diverse sources, subject to atmospheric transformation, and varying over space and time. Three common air pollutants, particulate matter (PM), ozone, and nitrogen dioxide (NO2), are the focus of most monitoring programs, communication efforts, health impact assessments, and regulatory efforts. Air pollution can also be classified into pollution of outdoor/ambient or indoor origin, both of which have serious health effects [6].

## Air pollution impacts on human health

**Roughly one third of deaths from the leading NCDs (stroke, lung cancer, heart attacks, and chronic obstructive pulmonary disease) are due to air pollution. Twenty percent of all CVD deaths, more than 3 million annually, are caused by air pollution** [6].

Evidence for impact on cardiovascular disease is most consistent for PM, which is responsible for the vast majority of the disease burden via its impact on ischemic heart disease and stroke, as well as lung cancer, COPD, lower respiratory infections, type 2 diabetes, pregnancy outcomes and related infant mortality [8]. PM particles come in many sizes and can be made of hundreds of different chemicals. In the context of air pollution, the most commonly referenced types of particulate matter are PM2.5 (made of fine inhalable particles with a diameter that typically does not exceed 2.5 micrometers) and PM10 (similarly inhalable particles with a diameter between 2.5 and 10 micrometers) [9].

Ozone (O_3_) is mainly associated with exacerbation of respiratory disease, COPD incidence and mortality. Nitrogen Dioxide, or NO_2_, is produced from the burning of fossil fuels and is often used as an indicator of traffic-related air pollution. There is growing evidence that chronic NO2 exposure is associated with effects on mortality, including cardiovascular deaths. Chronic exposure to NO2 is also associated with incident childhood asthma, while short-term variability is associated with exacerbation of asthma and increased daily mortality counts [11].

It is important to note that certain populations are at higher risk from air pollution. These populations can be classified as susceptible, vulnerable, or both. Susceptible groups are those at higher risk of cardiovascular events for a given level of pollution exposure. Cohort studies demonstrate that more susceptible populations include adults over 60, socio-economically disadvantaged and minority groups, and individuals with obesity, diabetes, hypertension, pulmonary disease, or atherosclerotic cardiovascular disease [12,13,14,15,16,17].

Vulnerable individuals are those exposed to elevated levels of air pollution. Important factors include living in regions of high pollution and proximity to any of the following: urban industrial emissions, heavy traffic, wildfires, seasonal agricultural burning, or burning of solid fuels for cooking or heating [12,17,18,19].

**Figure 1:**
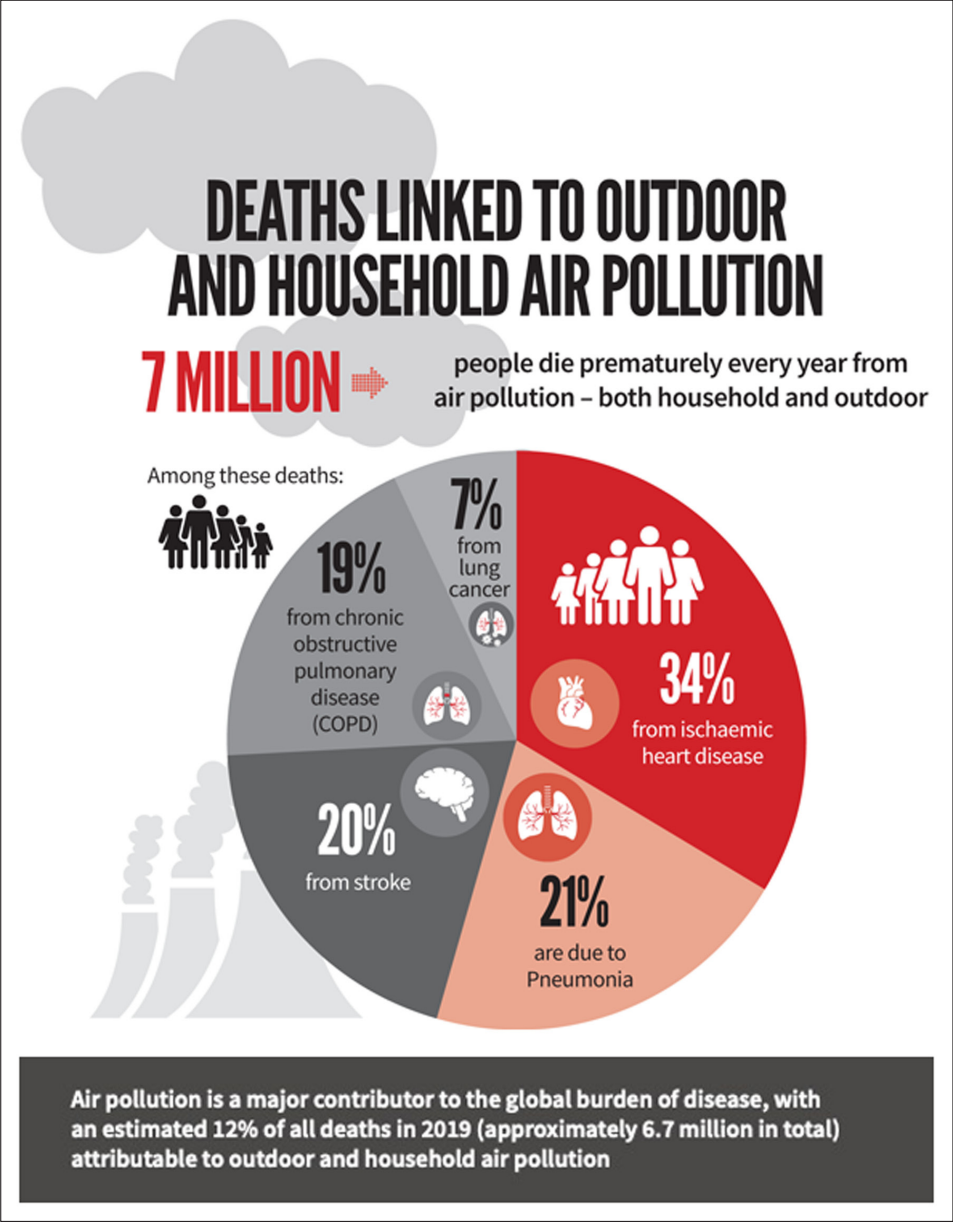
[10].

**Figure:**
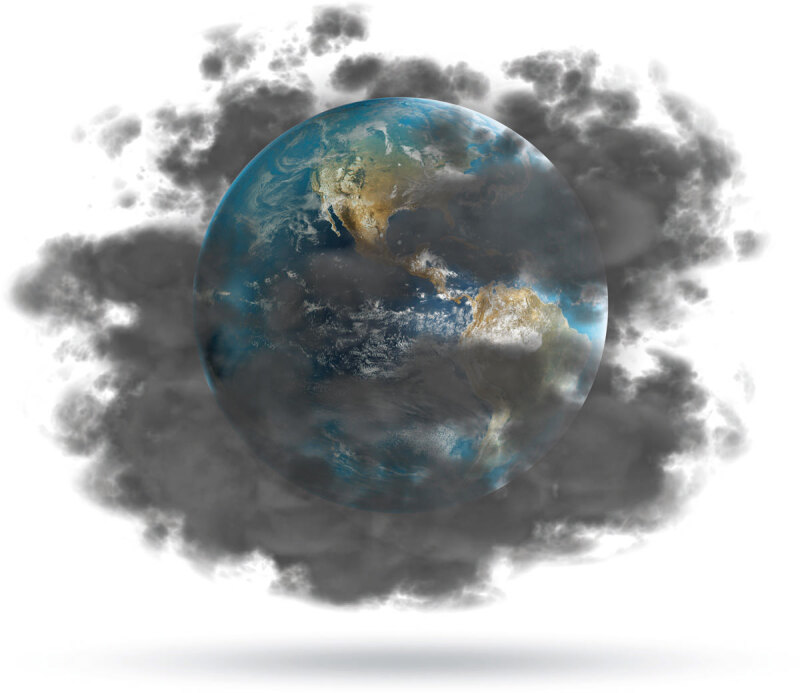


## Air pollution and cardiovascular disease

**Figure 2:**
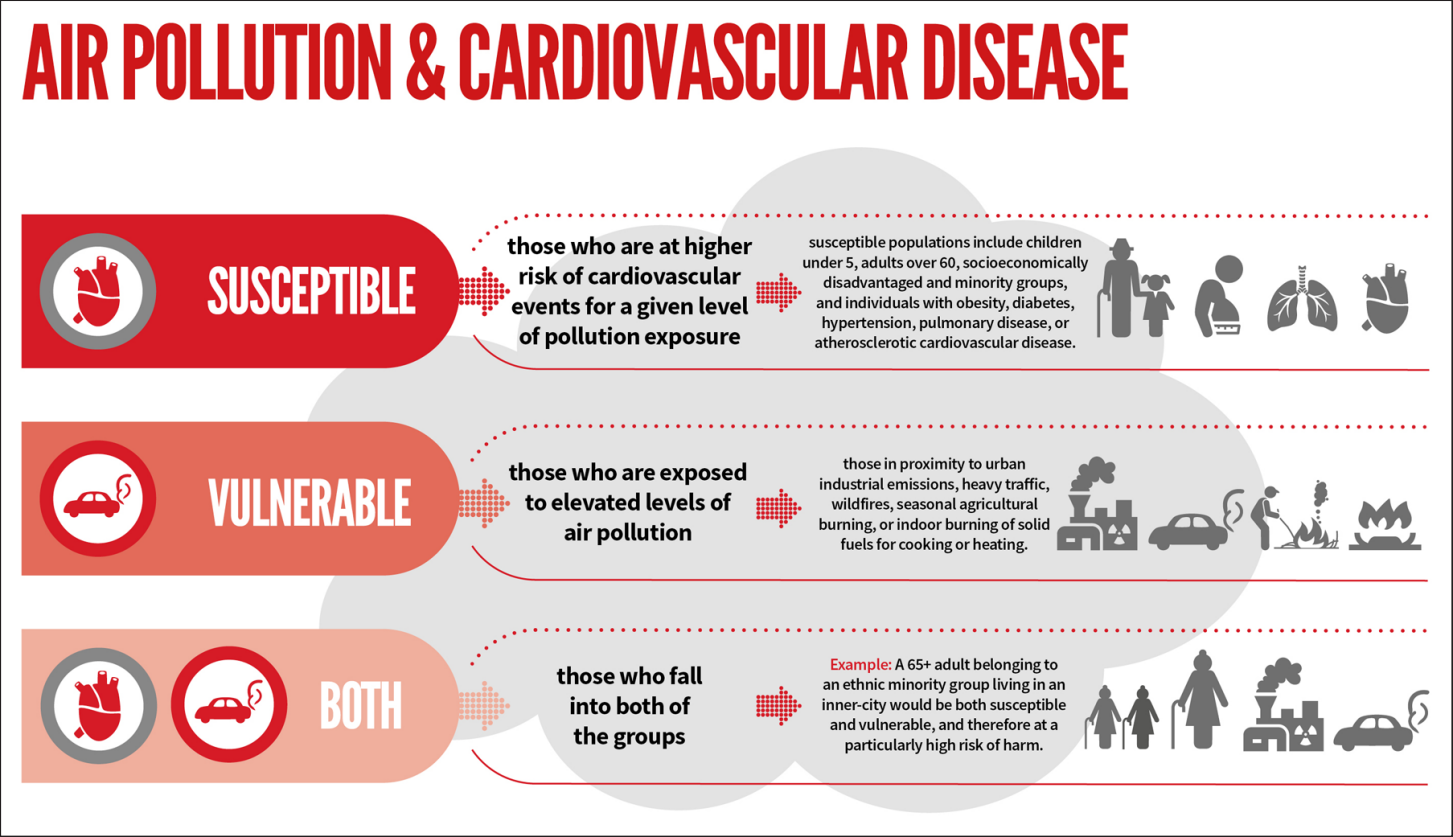
Susceptible and Vulnerable Populations.

**Figure:**
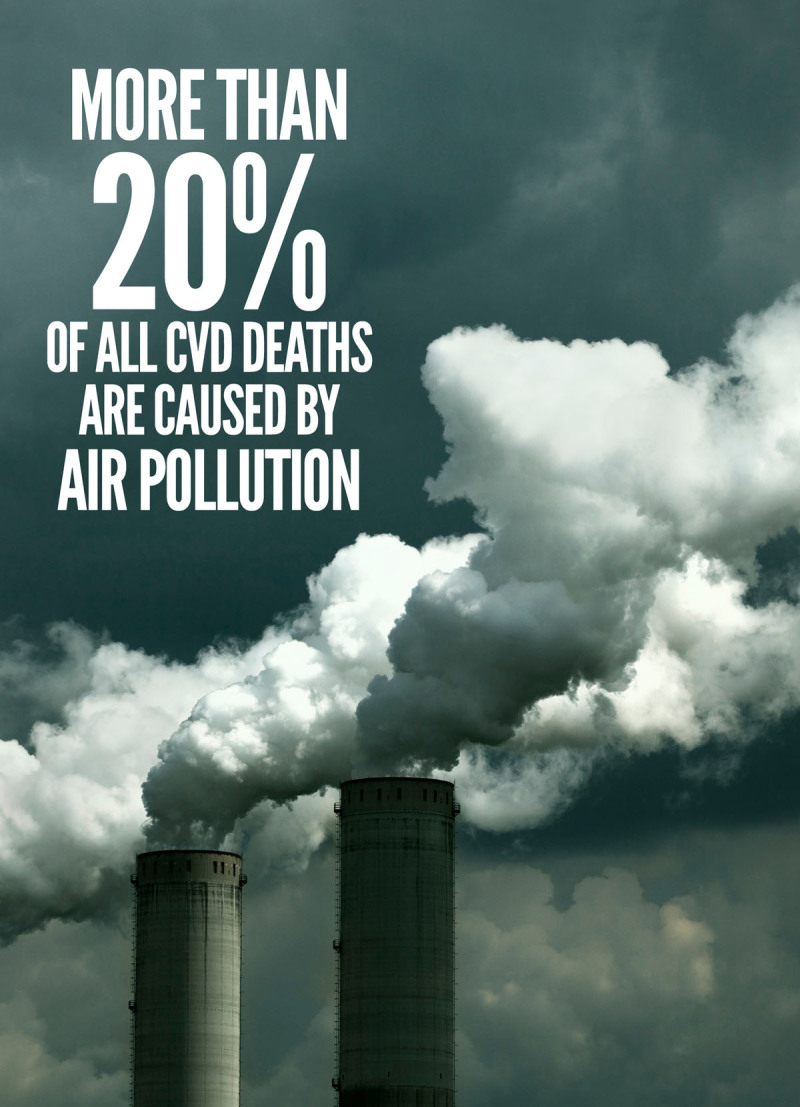


## The impact of air pollution on cardiovascular disease and the role of the global CVD community

**The World Heart Federation led the January 2021 publication of a Joint Opinion with the American College of Cardiology, American Heart Association, and the European Society of Cardiology: Taking a Stand Against Air Pollution – The Impact on Cardiovascular Disease** [20].

This initiative was based on the fact that while the impacts of air pollution on respiratory diseases is widely recognized, 50% of the estimated 6.7 million deaths attributable to air pollution in 2019 are due to cardiovascular diseases, and globally more than 20% of cardiovascular disease deaths were attributable to air pollution, with a disproportionate burden borne by low- and middle-income countries [11].

**Figure 3:**
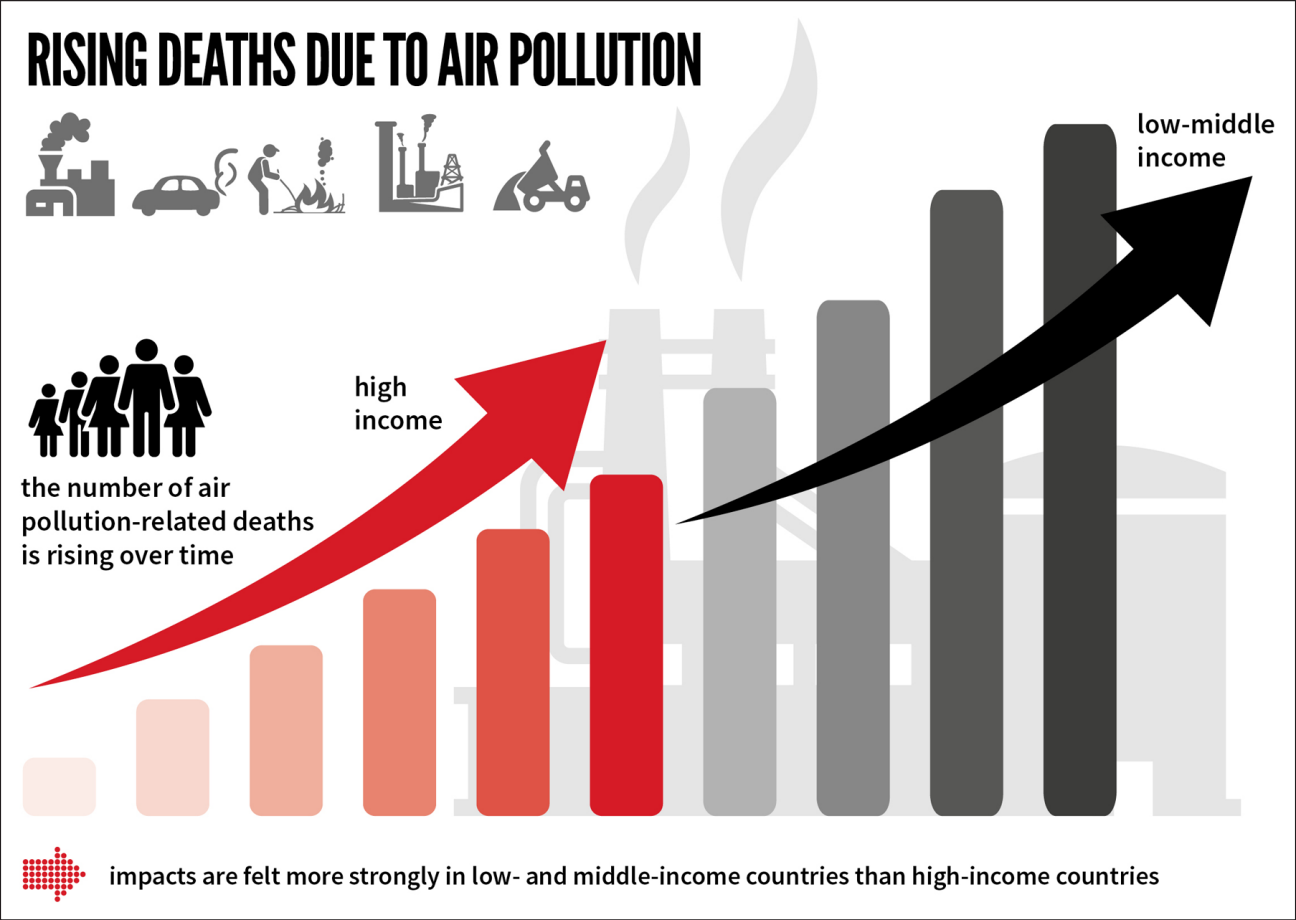
Impact of Air Pollution on Human Health.

**Figure:**
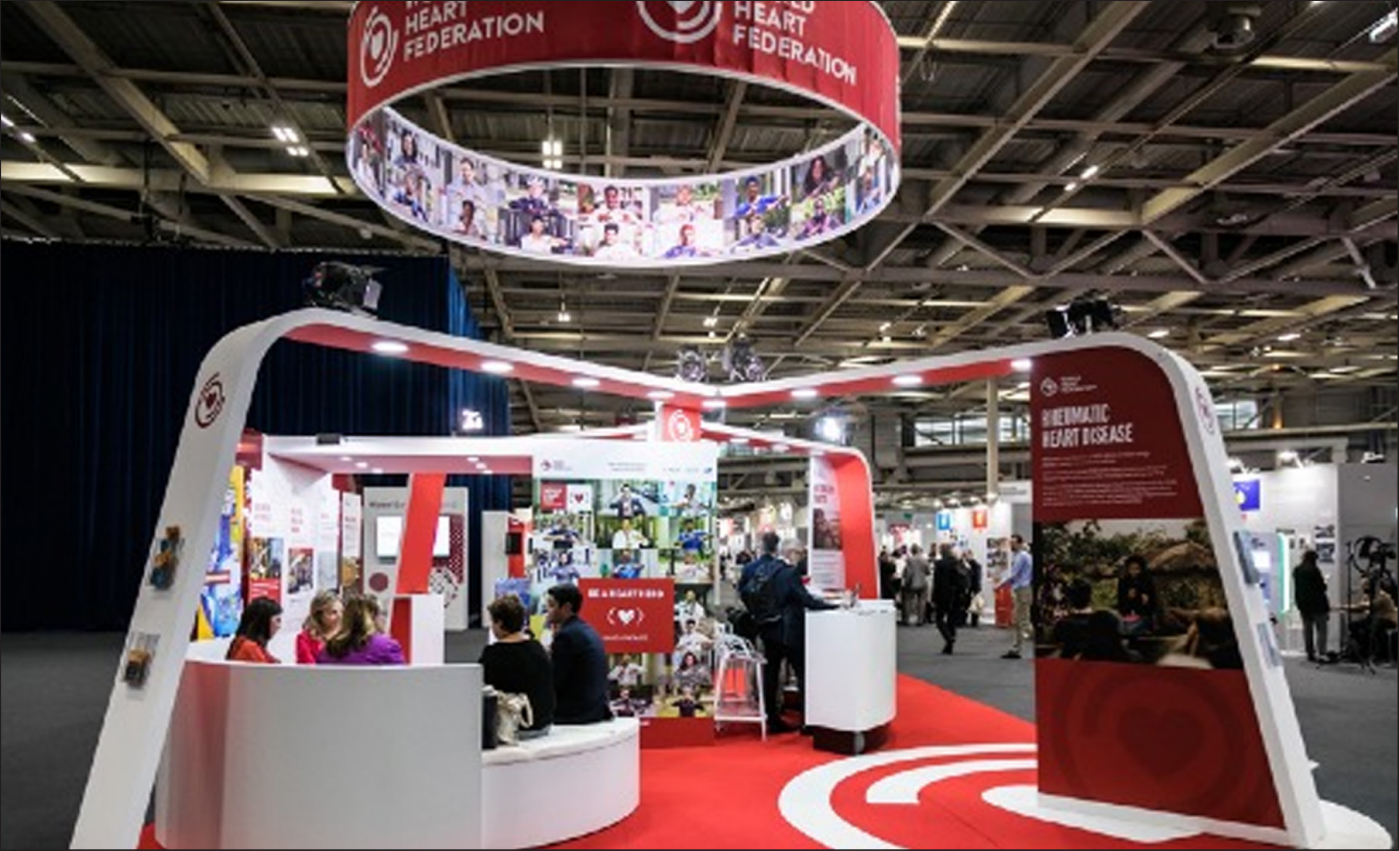


Overall, there is still a limited understanding amongst specialist physician and cardiologists of the importance of air pollution contributing to premature cardiovascular death [21]. With the position statement and further projects such as this document, WHF is committed to support research, advocacy, and education to reduce the impacts of air pollution on cardiovascular health. Specifically, WHF will disseminate new findings to its membership and via other activities, such as World Heart Day.

WHF with its members will educate and raise awareness among health care providers on the importance of reducing air pollution and the cardiovascular benefits of air pollution mitigation. WHF is working with senior decision-makers in national, regional and global governmental institutions to make air pollution related heart disease a priority and to identify interventions to reduce air pollution and its impact on NCDs. A number of projects are currently underway.

## Addressing the global CVD community

**The complexity and scale of this issue creates an unfortunate lack of understanding among those with the power to make change for good, including doctors and policymakers, which in turn results in a subsequent lack of concerted action.**

**Figure:**
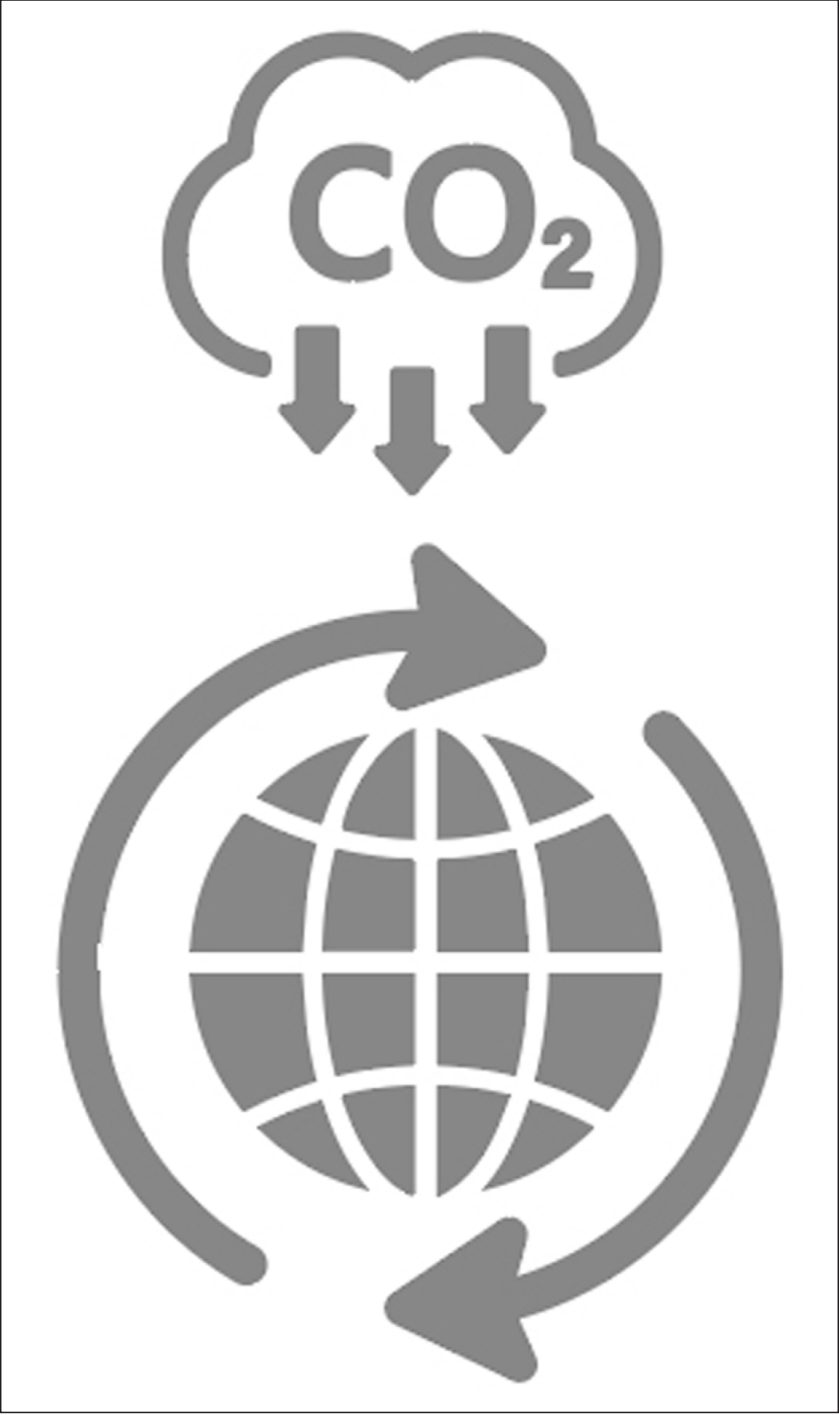


Indeed, political commitments and policy measures to mitigate pollution emissions will ultimately be necessary to reduce harmful exposures. Nevertheless, healthcare providers can play several important roles before, and while, such mitigation is achieved. WHF is ideally poised to address the low levels of acknowledgment and acceptance of the impacts of air pollution on circulatory health among cardiology societies, heart-health foundations, and institutes of medical education and their members working on the front lines of healthcare and health policymaking.

## Member survey

**In 2020, the World Heart Federation conducted a survey of its 200+ Members around the world to help assess the global cardiology community’s perception of the relationship between air pollution and CVD and better understand where WHF can make the most impact with its advocacy efforts.**

The results simultaneously indicated a strong interest in learning about and taking action on air pollution as well as concerning gaps in knowledge and limited awareness of relevant tools or educational materials (see Annex).

For example, of the 146 survey respondents, 141 either agreed or strongly agreed with the statement ‘Exposure to air pollution is one of a number of major risk factors for CVD’, but only 70 (less than 50%) felt that they have access to any tools or information they might need to better inform themselves and others about air pollution and CVD.

The survey nevertheless provided WHF with a clear mandate to consolidate the existing scientific evidence of the effects of air pollution on cardiovascular health and provide its Membership with a clear set of policy recommendations.

**A strong mandate for action:** *WHF members’ response when asked whether the world heart federation should publish and advocate for policies addressing air pollution and health.*

**Figure:**
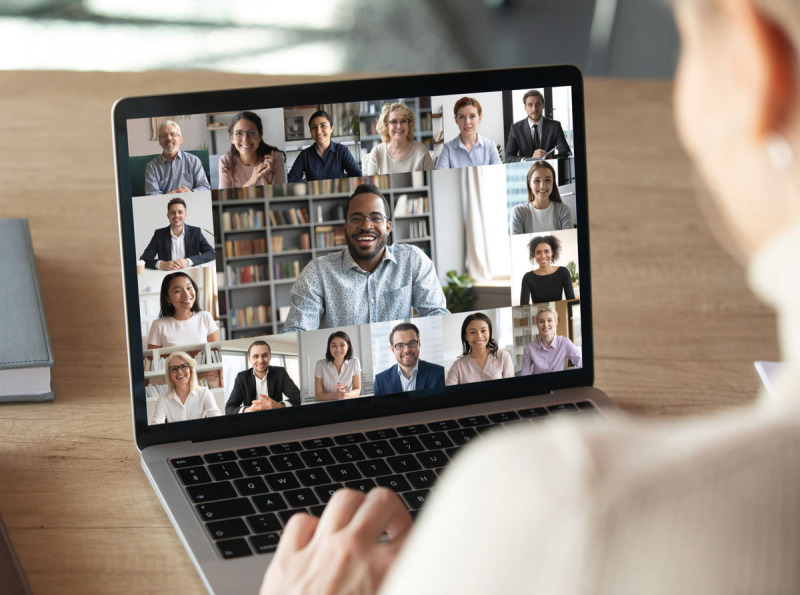


**Figure 4:**
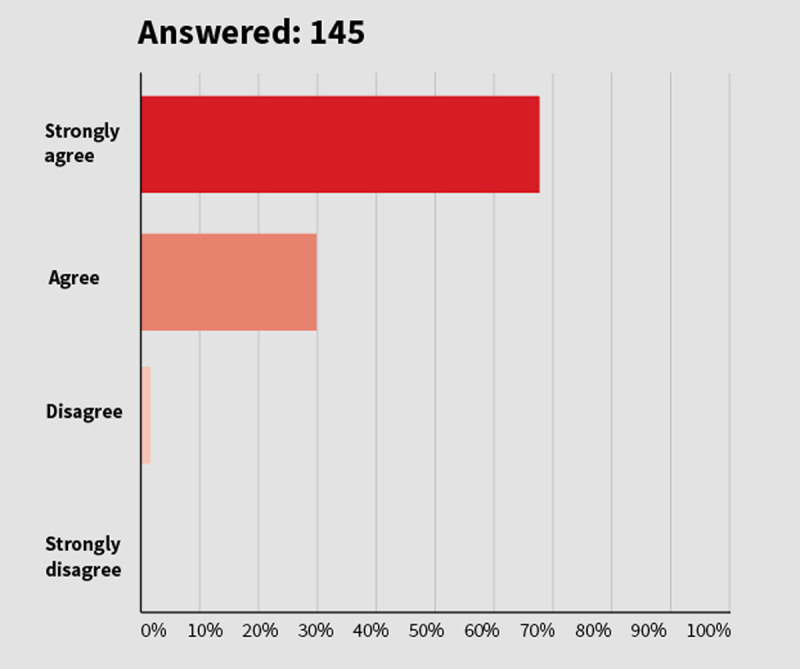
Results from Question 10 of WHF Members’ Survey.

## Mechanisms

**Figure 5:**
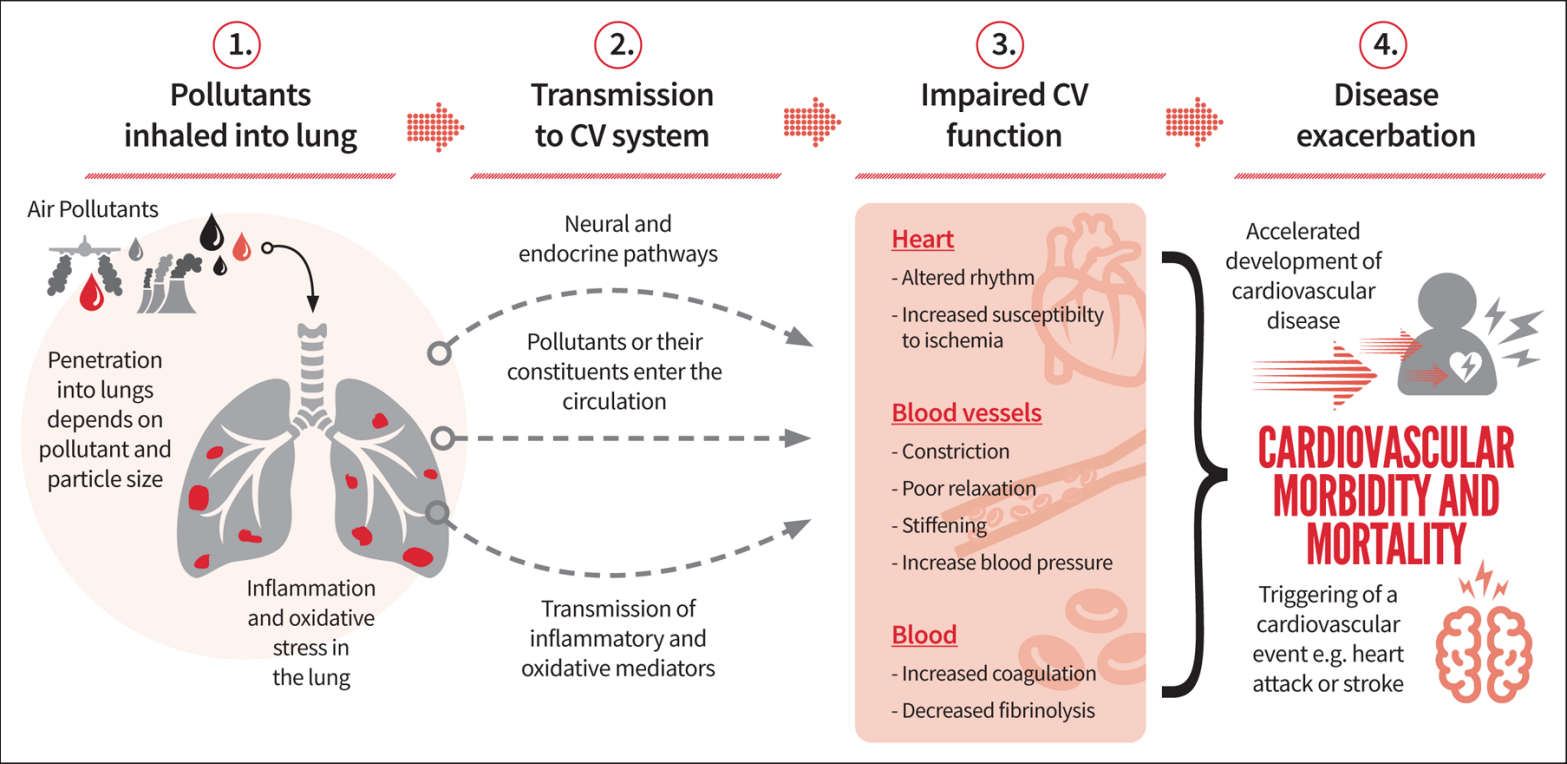
The biological mechanisms by which inhaled pollutants can cause cardiovascular (CV) morbidity and mortality [22].

There is now a wealth of epidemiological evidence showing that a number of air pollutants are associated with cardiovascular morbidity and mortality. This evidence is robustly supported with an equal amount of mechanistic work in cells, animal models and volunteer studies. Together they demonstrate plausible biological mechanisms to account for epidemiological associations. A network of interacting pathways connects inhalation of pollutants to cardiovascular morbidity. These are described below, and in Figure 5.

**Figure:**
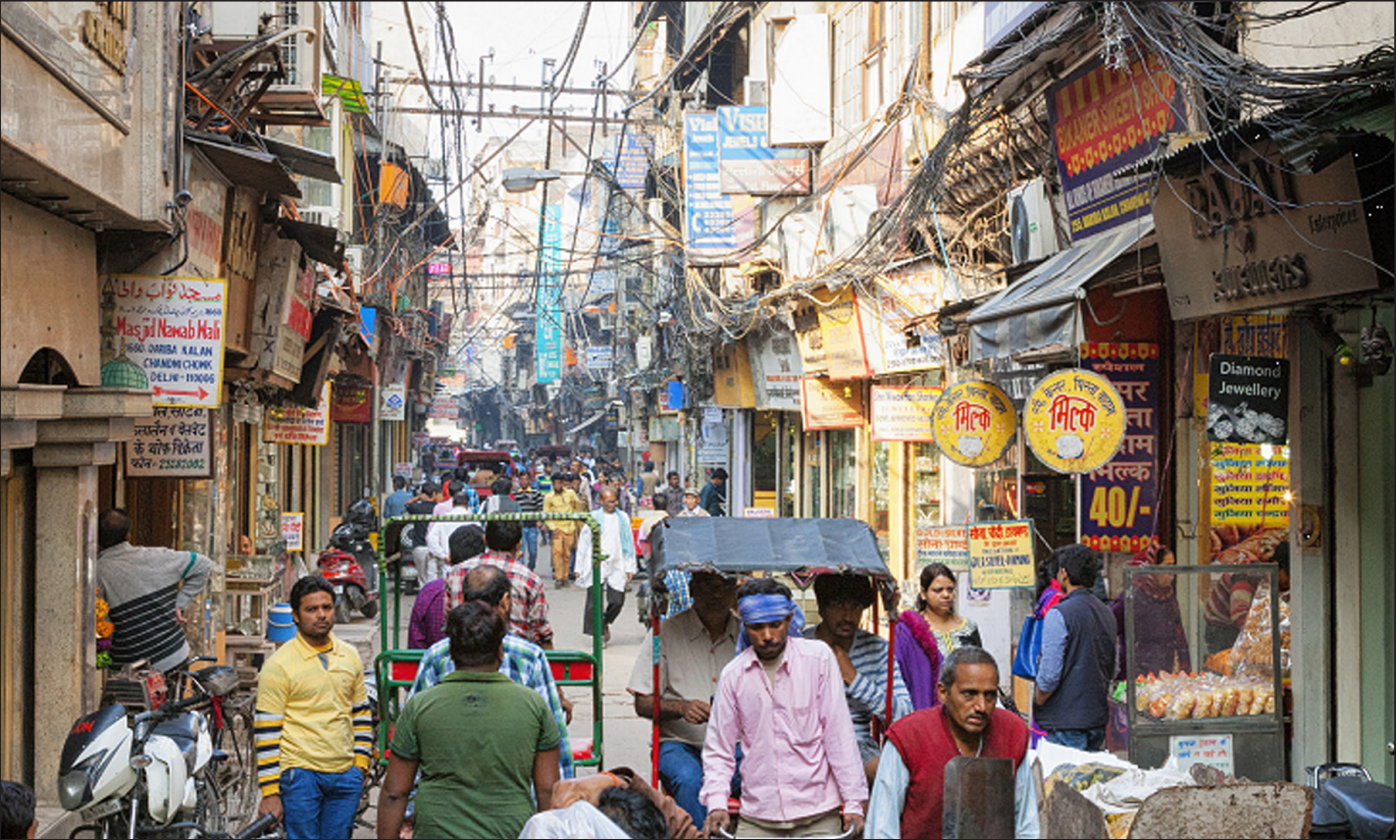


## Phases 1 & 2: Inhalation of pollutants and transmission to the cadiovascular system

Some gases such as ozone exert the majority of their effects in the upper airways, whereas other pollutants, such as nitrogen dioxide and fine particles, reach the air sacks of the lung. Many pollutants can induce inflammation and oxidative damage in the lung, the mediators of which pass into the circulation to cause adverse effects on the cardiovascular system. There is increasing evidence that small particles, or their constituents, can also pass into the circulation. Additionally, air pollutants may alter cardiovascular function through changes in neural activity and circulating blood factors. It is likely that several of the above pathways act in concert to induce different aspects of cardiovascular dysfunction and on different time scales.

## Phase 3: Cardiovascular function is impaired in many different ways

At the level of the cardiovascular system, air pollutants have a dizzying array of actions by which they impair cardiovascular function.

Effects include:

- Contraction and stiffening of arteries, as well as a decrease in the ability of blood vessels to relax,
- Increased blood pressure,
- Changes to heart rate and rhythm,
- Increased susceptibility of the heart to ischemic damage,
- Increased coagulability of the blood (i.e., the blood clots more easily) and decreased fibrinolysis (causing an inability to breakdown of clots to prevent excessive coagulation),
- Inflammation of blood vessels,
- Acceleration of the growth and development of atherosclerosis (the formation of fatty plaques form on the inner layer of arteries that is characteristic of coronary heart disease),
- Increased vulnerability of atherosclerotic plaques, making them more likely to rupture and block blood vessels.

## Phase 4: Changes to Cardiovascular function lead to a ‘cardiovascular event’

The actions listed above will detrimentally affect cardiovascular function. In healthy individuals, these actions would be expected to promote the risk of developing cardiovascular disease. In patients that already have cardiovascular disease, the effects could potentially trigger a cardiovascular event such as a heart attack or stroke [23].

Our increased understanding of the biological mechanisms by which air pollutants affect cardiovascular function explains, and greatly strengthens, the observations that air pollutants cause cardiovascular morbidity and morbidity. Moreover, the mechanisms highlight that the cardiovascular effects of air pollution are of concern to everyone, whether young or old, healthy or a person with existing disease.

**Figure:**
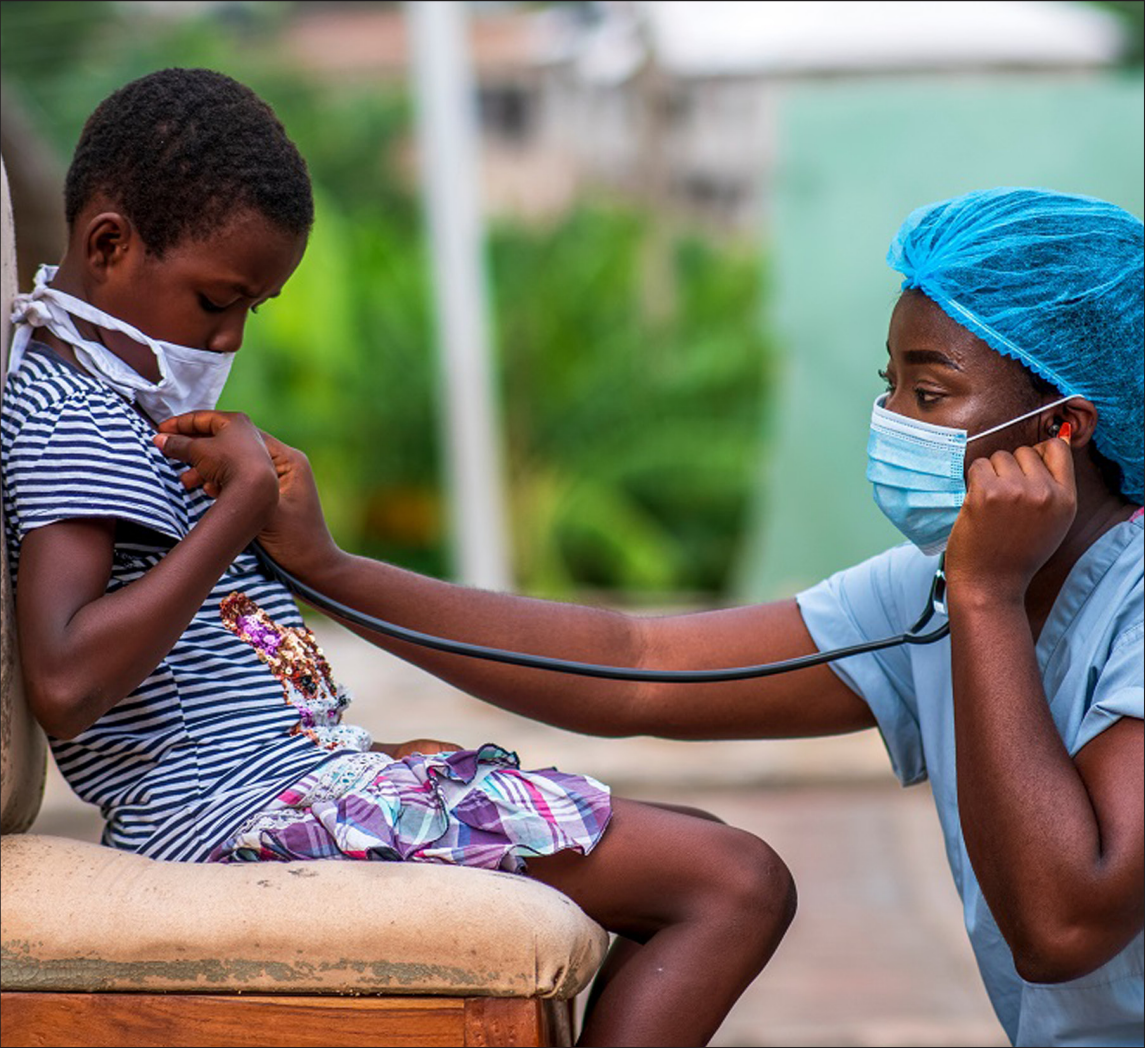
Niel Y. Shutterstock; 2020 [24].

## Multimorbidities: Aligning with the NCD agenda

**The mechanisms of harm outlined above demonstrate how the cardiovascular system bears the brunt of the negative impact of air pollution on human health. Yet air pollution is also a major risk factor for all of the leading causes of NCD mortality (including stroke, lung cancer, heart attacks and chronic obstructive pulmonary disease, with additional morbidities currently undergoing study).**

**Figure 6:**
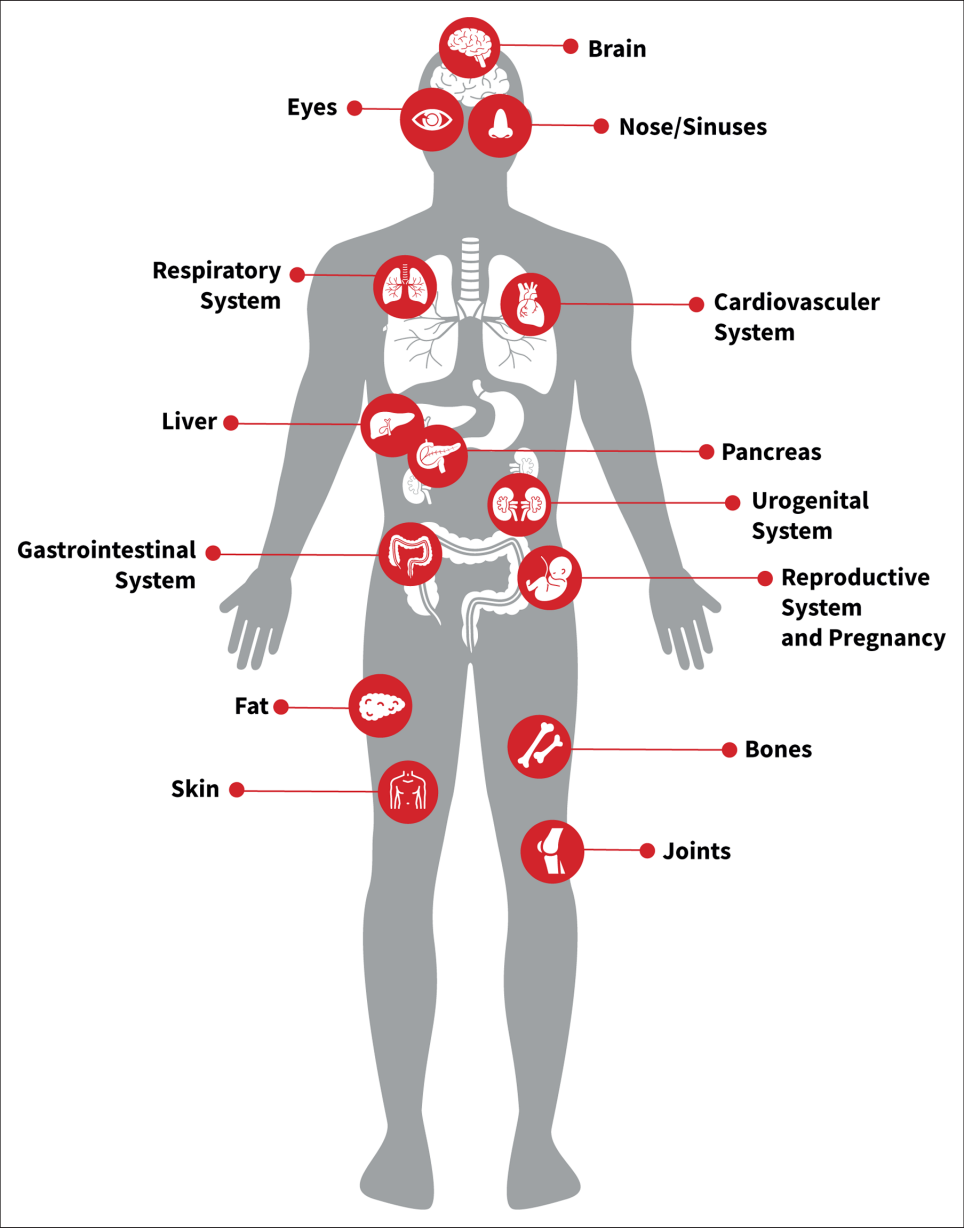
Impacts of Air Pollution on Body Systems [26].

The third United Nations High-Level Meeting on NCDs in 2018 went so far as to transform the “4 × 4” into a “5 × 5 agenda”, adding environmental air pollution as one of the five most important risk factors for the five deadliest non-communicable diseases [25]. With the growing recognition that air pollution can cause harm to many, if not all, organs of the body (Figure 6), and is associated with diseases in these organs such as diabetes and chronic kidney disease, it is likely that we are still underestimating the levels of morbidity and mortality caused by air pollution.

It is therefore essential that medical federations like WHF work across disease siloes with partners in the NCD space. Together these organizations, societies, and foundations, such as those comprising the Global Coalition for Circulatory Health and the NCD Alliance, can reach tens or even hundreds of thousands of physicians, advocates and members of the healthcare workforce around the world; all of whom have a part to play in the reduction of PM impacts on health. The convergence of other sectors and movements with a relevant role – from the energy industry to ministries of transportation, sustainable development think-tanks to mayors and local policymakers – contributes to the unique opportunity for action presented in the post-COVID era.

**Figure:**
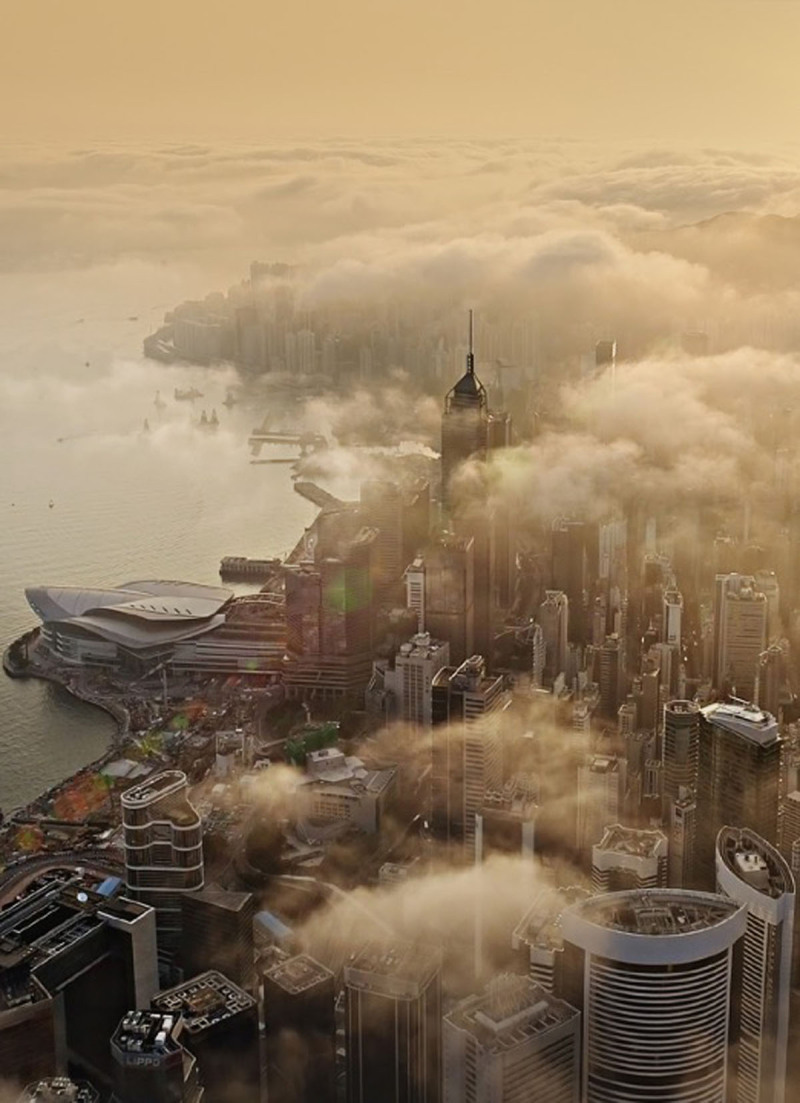


## Action on air pollution: from the individual to the global

**Clinicians can assess air pollution exposures for individuals using qualitative or quantitative approaches.**

Qualitatively, clinicians can ask their patients about established predictors of pollution exposures. A formal clinical screening tool has been proposed to identify these exposures but requires validation [27]. Quantitative estimation of pollution exposures requires greater sophistication but is increasingly available to healthcare providers. Many exposure models are publicly available and can be used to identify hazardous exposures in the community [27,28,29]. Finally, wearable pollution detectors may someday be incorporated into cellular phones and wristwatches, although such monitors still face challenges with calibration, maintenance, and stability [30,31].

However, it is clear that the negative effects of air pollution cannot be fully countered one patient at a time. Addressing air pollution effectively will require a multi-system and multi-sectoral response, and mayors and city-level policymakers are some of those best positions to enact effective measures. Cities often contain the highest concentrations of susceptible and vulnerable individuals. This is due in part to the collocation of hazardous pollution exposures and minority groups [32,33]. In the United States, for example, Black and Hispanic/Latino individuals with low socioeconomic status are exposed to higher pollution levels, due to the location of communities near heavy traffic and industrial sources [34,35]. Black and Hispanic/Latino individuals, as well as individuals with lower socioeconomic status, also have a greater risk of mortality attributable to air pollution than do non-Hispanic White individuals [14,15].

**Figure 7:**
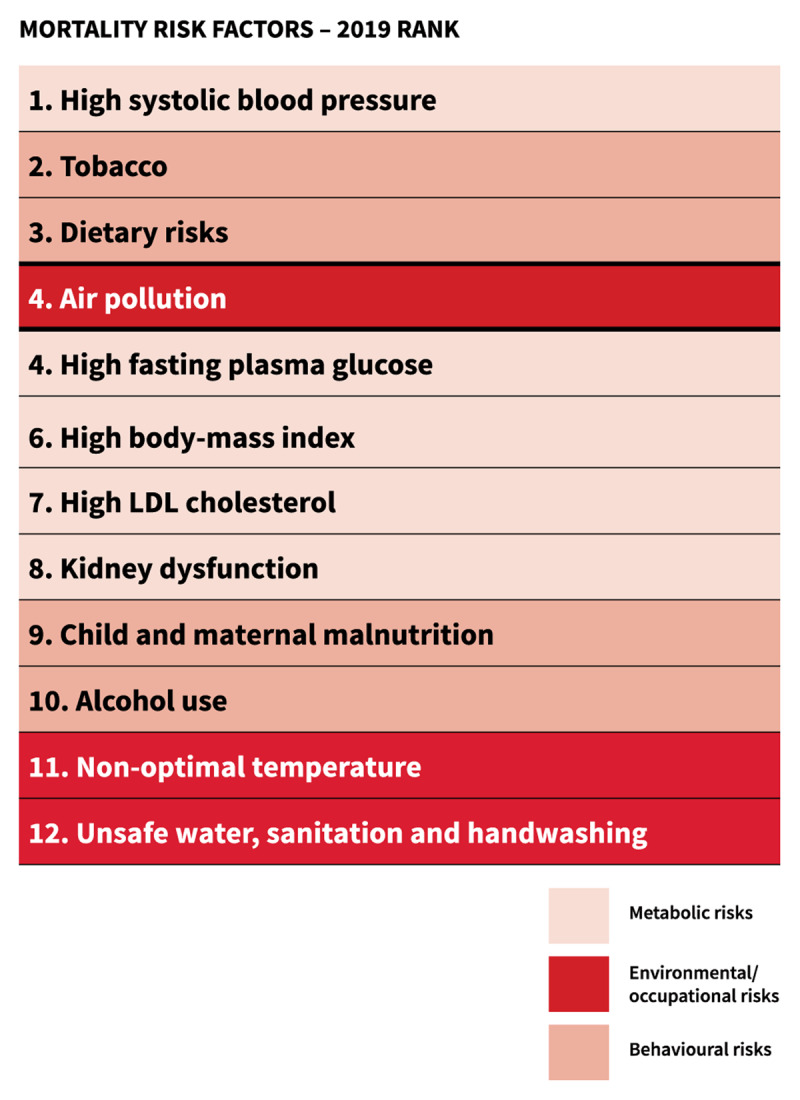
Global Mortality Risk Factors, 2019 Ranking [20].

The international community also has a key role to play in the mitigation of air pollution. Just as air pollution ignores national boundaries, so too has the global climate change movement grown beyond borders to capture the time and dedication of generations. The will of such movements has gone far and can continue to inspire tangible action such as the 2015 Paris Climate Agreement, which in turn has led to implementation of concrete policy at the national level [36]. Finally, States continue to exercise significant power in agenda setting through international fora such as UN High-Level Meetings and the World Health Assembly. There remains a significant opportunity for speaking strongly on air pollution in health policy spaces and likewise further integrating health impacts into climate change and air quality negotiations.

## Taking action: WHF Recommendations

**Having outlined the population- and individual-level cardiovascular risks associated with air pollution, the World Heart Federation recommends the following interventions for key stakeholders, with a sustained focus on cross-sectoral collaboration.**

**Table:** 

Target group	Action Items	Relative Strength of Evidence	Justification	Objective

**Physicians (I)**	*Use risk assessments to identify patients likely to benefit from interventions to reduce air pollution exposures, screening for susceptibility and vulnerability*. Personal measures may be necessary to reduce pollution exposures, particularly as reductions in ambient air pollution are unlikely in the short-term for much of the world; facemasks, particularly properly fit *N95 respirators, can block the majority of PM2.5 inhalation* [37].	**▲▲▲**	The effects of air pollution on the cardiovascular system are quantifiable and modifiable at the individual level [38]. Reducing air pollution exposures decreases the risk of cardiovascular mortality, acute coronary syndrome, stroke, arrhythmias, heart failure, and atherosclerosis [37]. Clinicians promoting cardiovascular health therefore have an opportunity and responsibility to protect their patients from air pollution.	Physicians and patients become aware of, and empowered, to ameliorate the impacts of air pollution on health. Personal measures are necessary to reduce pollution exposures, particularly as reductions in ambient air pollution continue at a slow rate in the short-term for much of the world [37]. Specific measures include personal masks, air filtration, clean stoves and fuels, behaviour modification, and dietary approaches. Although early trials have shown promising results on surrogate endpoints, large randomized trials are needed to evaluate the efficacy of these, and pharmacologic, interventions on preventing cardiovascular events. As we await additional data, clinicians can recommend these interventions to their most susceptible and vulnerable patients.
	Outdoor air pollution often infiltrates buildings, leading to hazardous indoor exposures [38]. *High-efficiency particulate air (HEPA) filters can remove the majority of indoor PM2.5* [17,37,39]. For households burning fossil fuels (e.g., oil, coal) or biomass (e.g., wood, dung) for cooking or heating indoors, key interventions include ventilation, electrification, and access to clean stoves and clean fuels [40]. *Portable HEPA air purifiers can also be considered in cases where preferred interventions cannot be achieved at scale*.	**▲▲**		
	*Behavioural modifications are a simple strategy to reduce air pollution exposures*. Advise susceptible individuals to stay indoors and close windows on days with elevated ambient pollution levels [38]. Where outdoor air pollution is low, windows can be opened to ventilate indoor environments. *Patients can be notified of daily levels via air quality alert networks, which provide warnings and recommendations on how to minimize exposures* [18,37].	**▲▲**		
	Individuals exposed to vehicular emissions should be counselled to avoid rush hour transit, close/open vehicle windows, and use car air conditioning/purifiers [17,41,42,43]. *For susceptible individuals, high-intensity outdoor exercise should be delayed during heavy pollution conditions*.	**▲▲▲**		
	Clinicians can make additional *behavioural recommendations targeting pollution exposures identified in the patient history* [44,45]. Dietary and pharmaceutical interventions show promise but require further investigation. For example, small trials have demonstrated that antioxidants (e.g., vitamins C and E) and *omega-3 fatty acids may reduce oxidative stress and inflammation* attributed to air pollution exposures [46,47]. Likewise, a large prospective cohort demonstrated that a Mediterranean diet reduced cardiovascular mortality attributed to air pollution exposure, but it is premature to recommend pharmaceutical interventions at this time [48]. Optimising therapies to treat current cardiovascular conditions may also lessen the risk of air pollution triggering cardiovascular events, although more research is required. Finally, mitigation of traditional cardiovascular risk factors (e.g., hypertension, diabetes, obesity, atherosclerosis) *can reduce susceptibility to cardiovascular events attributed to air pollution exposures* [37].	**▲▲**		
Physicians (II)	In addition to direct patient care, clinicians and associated organizations should play a leadership role in reducing pollution exposures in their communities. First, medical facilities, which historically have contributed heavily to pollution emissions, should become *models for low emissions and renewable energy* [33]. Second, *medical education and training should now include the pathophysiology and management of pollution-attributable cardiopulmonary disease* [17]. Third, providers *should track pollution exposures affecting their service populations* [27]. Many exposure models are publicly available [28,29] and can be used to identify high-risk neighborhoods to target for intervention.	**▲▲**	Physicians can be powerful advocates for policy action on behalf of their patients [49]. Cardiologists and members of the global cardiology community are especially well placed to advocate for air pollution control measures due to their central role in mitigating its health impacts.	Stronger cross-sector coalitions for policies countering air pollution.
	Fourth, providers should *develop community-tailored programs* for such neighborhoods, including individual interventions (see Part I above), public education campaigns, public-private partnerships, and advocacy to local policymakers. Finally, physicians lend their *support to existing air pollution campaigns*, such as the Medics for Clean Air manifesto [50].			
Scientific societies	Scientific and specifically cardiology societies can take action on the air pollution agenda by providing *tools and support for their members to learn about concrete actions and policies* they can enact. They should also work to *feature air pollution through their various platforms*, such as on high-level panels at Congresses and events, thereby raising the profile and acceptance of air pollution as a modifiable risk factor for cardiovascular disease.	**▲▲▲**	Societies have the power and presence, through journals, publications, conferences, workshops, and other communications with their members, to raise the profile of air pollution as a CVD risk factor and encourage policy action.	Physicians in the global cardiovascular community are more receptive to taking action on air pollution.
Foundations and patient advocacy groups	Civil society groups should work with physicians to *advocate for measures at the city level* (i.e., active transit improvements, investment in non-combustion sources of energy and transport, stricter emissions controls – see below) to strengthen their arguments and coalitions for policymakers. They can also *provide patients and civil society members with information about personal protection measures* (see Physicians I).		CVD and other patient advocates bring an important and compelling personal element to the ‘story’ of air pollution and health, as well as crucial knowledge of policy processes to facilitate concrete action.	Physicians, scientific societies, and patients are connected with policymakers and engaged in the policy process through civil society facilitation.
Policymakers at the city level	There are several key policies mayors and city-level policymakers can embrace to mitigate the harmful effects of air pollution. *Regulate emissions* to encourage the transition to clean energy sources through the implementation of Low Emission Zones, traffic charging and parking policies. *Zoning laws* should prevent the collocation of residential zones with industrial and traffic activities. Together with the promotion of *efficient public transportation networks and adequate infrastructure for walking/cycling*, this can help to encourage clean active transit and outdoor cardiovascular exercise among residents and commuters. Where possible, *building codes should be updated* to require indoor air filtration and fitting to reduce the penetration of ambient pollution. *Advisory and prevention monitoring* that reports to the community if a pollutant is exceeding health levels is important, and health care providers must receive adequate resources to identify and manage individuals at elevated risk of pollution-attributable cardiovascular disease (see Physician I and Scientific Society sections). Explore the use of taxes on fossil fuels and unhealthy commodities, penalties for excessive production of air polluting waste, various “green” incentives, and emission offsetting programmes as a means of financing and enforcing these policies. Finally, mayors and city-level policymakers should *work closely with physicians and members of the cardiovascular community* to implement such policies, ensuring broad community engagement and a focus on positive health impacts [50].	**▲**	Cities are often the nexus of highly polluted areas and those places where susceptible and vulnerable populations reside. Successful action at the city level can have the greatest impact on those with the greatest need [50]. Over the long term, addressing air pollution yields high return on investment for both citizen health and city budgets [51]. Furthermore, involving physicians in policy consultation provides scientific credibility and wider community buy-in.	Air pollution policies are implemented internationally at the level of global agreements and government ministers (especially of the environment, health, and transportation) create a foundation for continued collaboration.
National governments; policymakers at the global level (partici- pating in WHA, G10, COP, etc.)	Member State representatives active in global policymaking should work closely with physicians and members of the global cardiovascular community to implement air pollution policies that put health at the center, such as the WHO guidelines on mitigation of indoor air pollution. Through forums such as the World Health Assembly (resolutions), UN High-Level Meetings (political declarations), COP Summits, etc, national governments can demonstrate domestic successes in countering air pollution and keep emission control firmly on the international agenda.	**▲▲▲**	Involving physicians in the policy consultation process provides scientific credibility. Addressing air pollution through concrete policy yields high ROI for health and budgets [51]. Strong domestic and global policies on air pollution will respond to international citizen demonstrations demanding climate action.	Air pollution policies are implemented internationally at the level of global agreements and government ministers (especially of the environment, health, and transportation) create a foundation for continued collaboration.

KEY: ▲▲▲ **Intervention is recommended**. This intervention is evidence-based, low-risk to individuals, and feasible from a resource perspective. ▲▲ **Intervention should be considered**. This intervention has a growing evidence base, but may pose some challenges from a resource perspective. ▲ **Intervention may be considered for specific groups**, but requires further evidence before recommending to broader populations.

## Additional areas for research and consideration

Research is needed on several fronts to guide interventions to reduce the global burden of pollution-attributable cardiovascular disease.

Firstly, further work is needed to identify which sources of air pollution cause the greatest adverse effects in specific locations. PM is considered to have the greatest effect on human health, however, PM itself is complex mixture of tens of thousands of different chemical species derive from diverse sources. Additionally, a number of under appreciated sources are gaining attention, such as those from agriculture, non-exhaust traffic particles (tire, break and road wear), other transport sources (e.g., shipping and aviation) and indoor air pollution (given the high proportion of time we spend in indoor environments). Understanding the chemical nature of these pollutants, and the means by which they exert harm in the body, will provide important information to prioritise ways to reduce exposure to the most harmful pollutants.

Secondly, randomized trials are needed to test the efficacy of interventions on reducing specific cardiovascular outcomes like death, stroke, and myocardial infarction. Key interventions to test include: N95 respirators, HEPA air filters, cardioprotective medications, clean burning stove-fuel combinations, avoidance behaviours, and warning systems. Trials should quantify individual exposures with personal monitoring devices and collect data on cost-effectiveness to guide policy decisions.

Thirdly, validated screening tools and/or biomarkers are needed to help clinicians identify high-risk individuals and communities. These tools should be integrated into a framework of personalized medicine that includes screening and management of other well-known cardiovascular risk factors.

Fourthly, greater clarity is needed on the exposure-response relationship for different pollutant sources (e.g., occupational exposures), types of pollutants (e.g., ultrafine PM [particles smaller than PM2.5 which are not measured by pollution monitoring networks]), health outcomes (e.g., stroke), and specific patient populations (e.g., minority groups).

Finally, additional analysis of the relative impacts of policy implementation is always welcome. Research is needed to determine what amount of reduction in air pollution exposure translates into cardiovascular benefit. This should consider how different interventions and policies may alter the balance of the pollutant mixture, i.e., some interventions will have a greater effect on specific pollutants than others or may even reduce the targeted pollutant while increasing levels of co-pollutants. Such studies may also help physicians and policymakers with limited resources prioritize the most effective responses.

**Figure:**
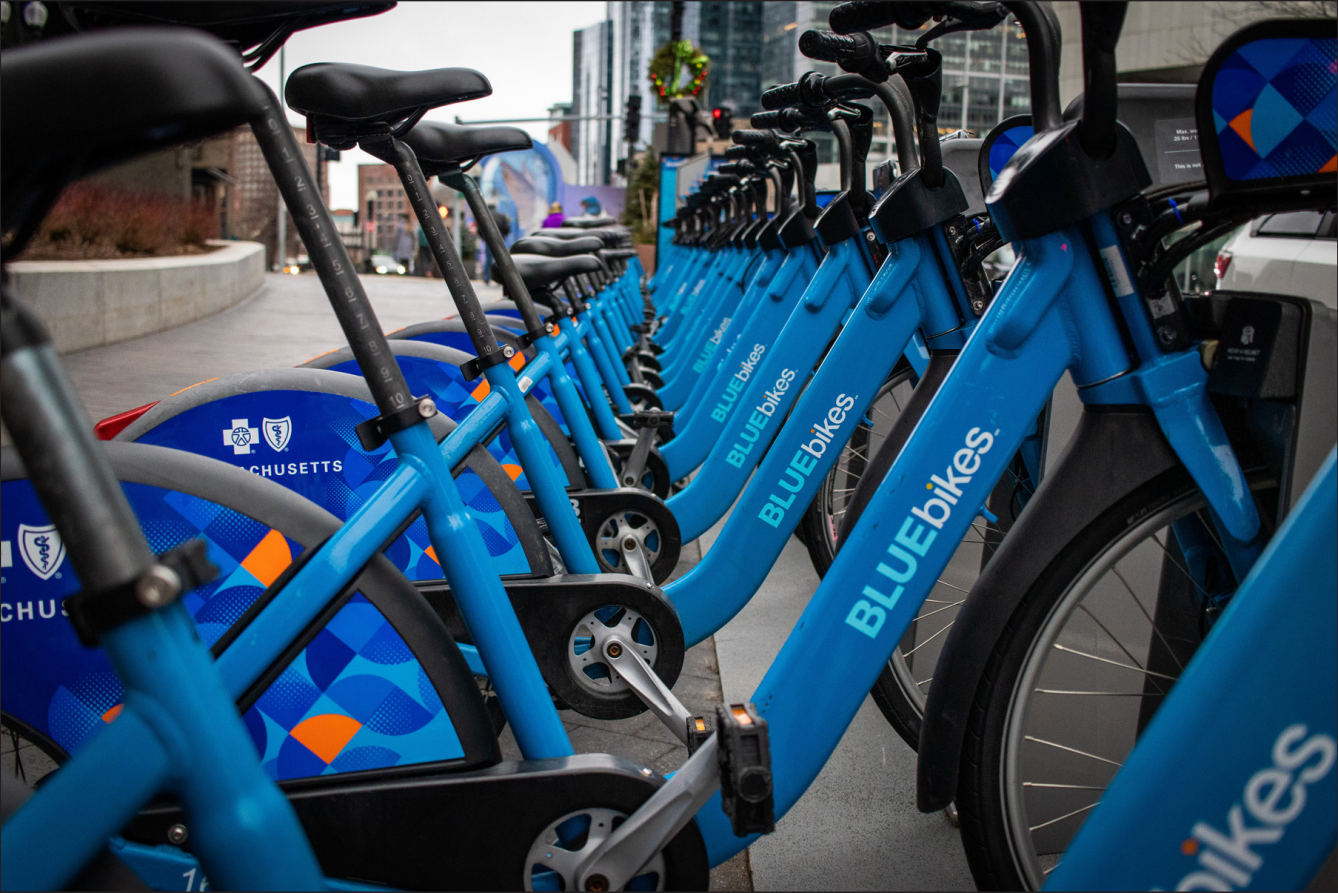
Doucett A. Unsplash; 2021 [52].

**Figure:**
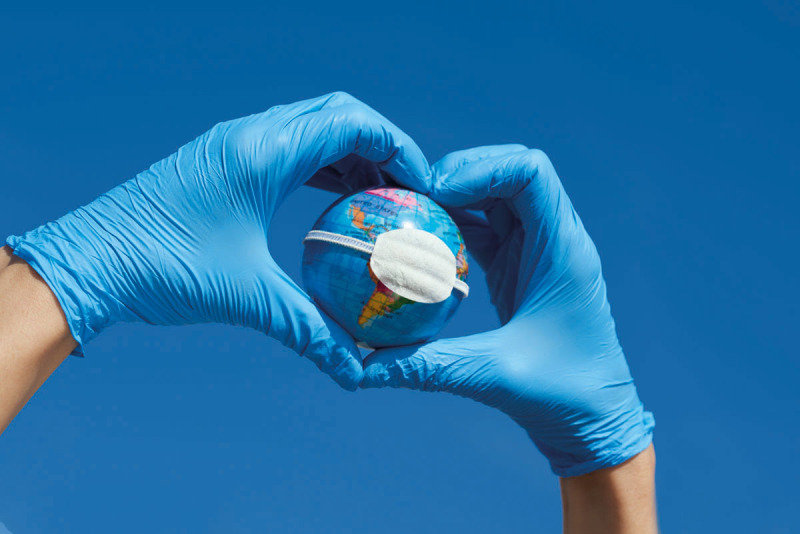


## The future of air pollution as an environmental determinant of health

**In 2019, air pollution was recognized as the fourth-highest ranking risk factor for mortality, with more attributable deaths than high LDL cholesterol, high body-mass index, physical inactivity, or alcohol use** [7].

Yet at the time of this publication in 2021, many physicians, scientific societies, advocates, and policymakers remain unaware of air pollution’s prominent position as a threat to health and continue to operate as if its manifold impacts are negligible, unmodifiable, or perhaps simply too overwhelming to confront. However, the momentum of international climate change movements and the dramatic impacts of the COVID-19 pandemic on human society are creating an unprecedented opportunity to revaluate the role of the global cardiovascular community in tackling this critical issue.

Through targeted patient interventions, key city-level infrastructure investments, and above all concrete policy action on an international scale, the morbidity and mortality associated with air pollution’s effects on the cardiovascular system can be significantly reduced. Lives can be saved, budgets balanced, and the virtuous cycle of sustainable urban mobility can pave the way for blue skies on the horizon.

## Author Information

WHF Air Pollution Expert Group:

**Chair – Prof Michael Brauer,** The University of British Columbia, Institute for Health Metrics and Evaluation (University of Washington) (Canada/United States)

**Dr Narantuya Davaakhuu,** National Center for Public Health Mongolia (Mongolia)

**Professor Maria Consuelo Escamilla Nuñez,** Instituto Nacional de Salud Pública (Mexico)

**Dr Michael Hadley,** Mount Sinai (United States)

**Mr Daniel Kass,** Vital Strategies (United States)

**Dr Mark Miller,** Centre for Cardiovascular Sciences, University of Edinburgh (United Kingdom)

**Professor Dorairaj Prabhakaran,** Public Health Foundation India (India)

**Professor Karen Sliwa,** Cape Heart Institute, Faculty of Health Sciences, University of Cape Town (South Africa)

**Dr Ta-Chen Su,** Department of Environmental and Occupational Medicine, National Taiwan University College of Medicine (Taiwan)

**Dr Ilonca C.H. Vaartjes,** Julius Center for Health Sciences and Primary Care, University Medical Center Utrecht (The Netherlands)

**Dr Rajesh Vedanthan,** NYU Grossman School of Medicine, New York (United States)

WHF Staff and Team:

**Kelcey Armstrong-Walenczak**

**Jeremiah Mwangi**
